# P96 Identification of novel KPC-2 mutations conferring resistance to β-lactam/β-lactamase inhibitor combinations: implications for genomic surveillance and clinical diagnostics

**DOI:** 10.1093/jacamr/dlaf230.103

**Published:** 2025-12-04

**Authors:** Hawa Ahmed, Maria Rosa Domingo-Sananes, Gareth McVicker, Alasdair T M Hubbard

**Affiliations:** School of Science and Technology, Nottingham Trent University, Nottingham, UK; School of Science and Technology, Nottingham Trent University, Nottingham, UK; School of Science and Technology, Nottingham Trent University, Nottingham, UK; School of Science and Technology, Nottingham Trent University, Nottingham, UK; School of Veterinary Medicine and Science, University of Nottingham, Nottingham, UK

## Abstract

**Background:**

β-Lactam/β-lactamase inhibitor combinations (BLICs) are commonly used in the UK and are a fertile area of drug development. BLICs such as ceftazidime/avibactam (CZA) and meropenem/vaborbactam (MEV) were developed as vital therapeutic options for KPC-producing carbapenem-resistant Enterobacterales (CRE). However, resistance to BLICs is increasingly reported, often mediated by mutations within β-lactamase genes that alter enzyme-inhibitor interactions. Currently, genomic surveillance fails to detect uncharacterized inhibitor-resistant mutants, leading to discordance between the phenotype and genotype. This limits our ability to predict treatment success and to select the most appropriate antibiotic, as well as limiting the treatment options available.

**Objectives:**

Using a molecular biology approach, we aimed to identify novel mutations in *bla*_KPC-2_ that confer resistance to CZA and MEV to improve resistance prediction using genomic data.

**Methods:**

We used random mutagenesis to generate a mutant library of *bla*_KPC-2_, cloned into the low-copy plasmid pBR322 and screened for resistance to CZA and MEV. We tested three concentrations: 2× MIC, 4× MIC and the clinical breakpoint. Resistant clones were extracted for whole-plasmid sequencing using Illumina short-read sequencing to identify single nucleotide polymorphisms (SNPs) within the gene. Mutations were then mapped to the WT *bla*_KPC-2_ and assessed for their potential impact on enzyme function bioinformatically.

**Results:**

We found that while random mutagenesis produced CZA-resistant mutations within *bla*_KPC-2_, we were unable to identify SNPs that confer resistance to MEV. The mutants exhibited resistance to 2× MIC; a subset also showed resistance to 4× MIC, and to the clinical breakpoint concentration. Analysis of 45 SNPs revealed that 58% were located outside the characterized active site loops, although there was notable clustering in the β3-β4 loop (5 SNPs), β5-α11 loop (4 SNPs) and omega loop (10 SNPs). Importantly, all mutagenesis reactions reproduced the clinically significant D178 mutations in the omega loop. Approximately 45 % (39) of the non-synonymous mutations identified 39 are predicted as intolerant, therefore affecting enzyme function, and 16 of which confer resistance at the clinical breakpoint. Additionally, comparison with β-lactamase databases showed that only 20% of the identified SNPs have been reported in clinical isolates, indicating that 80% represent novel mutations with potential clinical relevance for CZA resistance.

**Conclusions:**

We identified a substantial reservoir of uncharacterized *bla*_KPC-2_ mutations predicted to be capable of conferring resistance to CZA. These findings have immediate implications for genomic surveillance, diagnostics and antimicrobial stewardship, as incorporation of these novel variants into resistance databases will enhance the accuracy of molecular diagnostics and guide more effective antimicrobial prescribing. Future phenotypic characterization of these mutations through site-directed mutagenesis will provide essential data for improving resistance prediction.

